# Integrating single-cell transcriptomics and whole-genome CRISPR CAS9 screen identifies a cell cluster associated with tumor dependency in triple-negative breast cancer

**DOI:** 10.3389/fonc.2025.1705923

**Published:** 2025-10-17

**Authors:** Su Liu, Shuo Wang, Guixin Wang, Yingxi Li, Zhigang Zhao, Yao Tian, Junming Cao

**Affiliations:** ^1^ Department of Hematology, Tianjin Medical University Cancer Institute and Hospital, Tianjin Cancer Hospital Airport Hospital, National Clinical Research Center for Cancer, Key Laboratory of Cancer Prevention and Therapy, Tianjin’s Clinical Research Center for Cancer, Tianjin, China; ^2^ the First Department of Breast Cancer, Tianjin Medical University Cancer Institute and Hospital, National Clinical Research Center for Cancer, Tianjin, China; ^3^ Health Science Center, Ningbo University, Ningbo, Zhejiang, China; ^4^ Department of Medical Oncology, Tianjin First Central Hospital, School of Medicine, Nankai University, Tianjin, China; ^5^ Department of Thoracic Surgery, The Affiliated LiHuiLi Hospital of Ningbo University, Ningbo, Zhejiang, China

**Keywords:** triple-negative breast cancer, tumor dependency, ScRNA-seq, CRISPR-Cas9, cell proliferation

## Abstract

**Background:**

Triple-negative breast cancer (TNBC) is the most aggressive breast cancer subtype; however, clinically approved prognostic biomarkers and therapeutic options remain limited. This study aimed to investigate tumor dependency genes to identify novel therapeutic targets for TNBC.

**Methods:**

Tumor dependency genes for TNBC were identified using the The Cancer Dependency Map (DEPMAP) database. The TCGA-BRCA dataset was utilized to analyze the expression, survival associations, and pathway enrichment of these genes. Single-cell datasets were employed to explore cellular trajectories and biological functions within tumor dependency gene-associated cell subpopulations. Genomic sequencing was used to investigate the somatic mutational landscape influencing the infiltration abundance of the tumor dependency-associated subpopulation. The METABRIC dataset assessed the impact of the tumor dependency-associated subpopulation on radiotherapy, chemotherapy, and combination therapy outcomes. Potential drugs were identified using the Connectivity Map (CMAP). Colony formation experiment and the CCK-8 experiment were performed to validate the biological function of gene.

**Results:**

Four tumor dependency genes (TDGs) were identified. These genes were highly expressed in TNBC and associated with poor prognosis. Enrichment analysis revealed their significant involvement in cell cycle-related pathways. Single-cell analysis demonstrated that the tumor dependency-associated subpopulation (TDAS), defined by these four genes, resided at the differentiation terminus of epithelial/tumor cells and was linked to energy metabolism and cell proliferation pathways. Crucially, patients with high TDAS infiltration abundance were found to be unsuitable for surgery alone and should receive combined radiotherapy or chemotherapy. Potential therapeutic agents targeting the TDAS were screened. And *in vitro* experiments confirmed the cell proliferation role of candidate genes.

**Conclusion:**

This study identifies four potential TNBC biomarkers for assessing TDAS abundance, providing novel insights and strategies for personalized TNBC treatment.

## Introduction

Breast cancer is one of the most common malignancies in women, with triple-negative breast cancer (TNBC) being the poorest prognosis subtype (1, 2). Due to the lack of endocrine therapy targets, TNBC patients cannot benefit from targeted therapy or hormone therapy as in other subtypes (3). Moreover, heterogeneity within the tumor microenvironment (TME) among TNBC patients contributes to its high metastatic potential and resistance to treatment (4). Current researches have tried to focus on targeting tumor-associated cells within the TME, such as cancer-associated fibroblasts (CAFs), to identify genetic markers and therapeutic strategies tailored to specific patient populations (5, 6). In addition, classification systems based on gene expression profiles or immunohistochemical cell types have been developed to support subtype-specific therapeutic approaches (7, 8). However, it remains limited for clinically approved prognostic biomarkers and treatment options in TNBC, making personalized management and treatment of TNBC a continuing challenge (9). Therefore, identifying novel molecular subtypes and prognostic biomarkers in breast cancer is urgently needed to support patient stratification and enable more precise, targeted therapies.

The CRISPR-Cas9 system is currently one of the most widely used gene-editing technologies. By combining the Cas9 endonuclease with a single guide RNA (sgRNA), the system enables precise targeting of specific DNA sequences, inducing double-strand breaks that can be repaired through the homology-directed repair (HDR) pathway to achieve accurate gene editing (10). This system allows for targeted manipulation of individual genes, facilitating functional studies at the single-gene level (11). Moreover, CRISPR-Cas9 has been instrumental in generating disease models, particularly in mice, by introducing specific gene alterations associated with human diseases, thereby greatly advancing biomedical research (12). In the field of oncology, the CRISPR-Cas9 system has been broadly applied to investigate the roles of specific genes in tumor cell proliferation, migration, and drug resistance by enabling gene knockouts (13, 14). Importantly, CRISPR-based genome-wide screening allows for the systematic knockout of large numbers of genes to identify those essential for specific cancer-related biological processes (15). This approach facilitates the discovery of key molecular targets across different tumor types, providing valuable insights for mechanistic studies and the development of targeted therapies.

In this study, we integrated multi-omics data to identify key tumor cell subpopulations in TNBC that serve as prognostic markers and potential therapeutic targets. Using a CRISPR-Cas9-based screening approach, we identified genes with tumor dependency in TNBC tumor cells and characterized a subset of tumor cells. These cells, which exhibit elevated expression of the identified genes, may represent a terminally differentiated tumor cell state and are associated with poorer patient prognosis. Finally, we screened for potential therapeutic agents targeting these genes, providing a basis for personalized treatment strategies in TNBC.

## Methods

### Data acquisition

This study utilized comprehensive datasets, including single-cell RNA sequencing (scRNA-seq) data, bulk RNA sequencing (bulk RNA-seq) data, genome-wide CRISPR-Cas9 screening data, and genomic sequencing data. The scRNA-seq data, comprising samples from five TNBC patients, were obtained from the Gene Expression Omnibus (GEO) database under accession numbers GSE180286 and GSE199515. Bulk RNA-seq data for breast cancer were downloaded from the TCGA-BRCA project within The Cancer Genome Atlas (TCGA) database and the METABRIC cohort within the cBioPortal database. For subsequent analysis, only bulk RNA-seq samples identified as TNBC and possessing both transcriptomic data and associated follow-up information were included. Additionally, genomic sequencing data were retrieved from the TCGA database. And the genome-wide CRISPR-Cas9 screening data was downloaded by the DEPMAP database.

### Data preprocessing

CRISPR-Cas9 screening data from breast cancer cell lines, processed using the CERES algorithm, were extracted. Based on lineage subtyping, data from 22 TNBC-like cell lines (defined as *ER*-negative and *HER2*-negative) were included in subsequent analyses. Based on previous study (16), tumor dependency genes were identified according to the following criteria: 1. The mean CERES score across cell lines was < −1; 2. CERES scores were < −1 in at least 80% of the cell lines.

### Downstream analysis of bulk RNA sequencing

Differential expression analysis between normal breast tissue and TNBC samples from the TCGA dataset was performed using the limma package. Genes with |log_2_FC| > 0.5 and an adjusted *P*-value < 0.05 (FDR) were considered differentially expressed, with log_2_FC > 0.5 defining overexpressed genes. Univariate Cox proportional hazards regression analysis was conducted on the 666 tumor dependency genes using the survival R package. Genes with a *P*-value < 0.05 and a hazard ratio (HR) > 1 were considered statistically significant predictors of survival. Kaplan-Meier (KM) survival analysis was employed to evaluate the impact of expression levels of candidate genes (*TONSL*, *TIMELESS*, *RFC3*, *RAD51*) on overall survival (OS). A log-rank *P*-value < 0.05 was considered statistically significant.

Gene Set Enrichment Analysis (GSEA) was performed to identify signaling pathways associated with the four tumor dependency genes. Using *TONSL* as an example, the 121 TNBC samples were stratified into high- and low-*TONSL* expression groups based on the median *TONSL* expression value. Differential gene expression analysis between these groups was performed using limma. Genes with log_2_FC > 0 were considered upregulated in the high-*TONSL* group. The resulting differentially expressed genes were ranked by log_2_FC (descending order). GSEA was then executed using the clusterProfiler R package with the pre-ranked gene list. Reference gene sets included c2.cp.kegg_legacy.v2023.2.Hs.symbols.gmt (KEGG pathways) and genesets.v2023.1.hallmark.Hs.gmt (Hallmark gene sets), downloaded from the official GSEA website.

From the METABRIC cohort, 319 TNBC patients were stratified into four groups based on treatment history (1): No radiotherapy (RT) or chemotherapy (CT) (2), RT only (3), CT only, and (4) RT + CT. The tumor dependency gene signature score (representing the enrichment score of the C6 subpopulation) was calculated for each sample using single-sample Gene Set Enrichment Analysis (ssGSEA) implemented in the GSVA R package. The impact of the tumor dependency signature score on overall survival (OS) was assessed using optimal cut-point analysis (via the surv_cutpoint function in survminer).

### Somatic mutation analysis

Simple Nucleotide Variation (SNV) data were obtained using the TCGAbiolinks R package. TCGA-TNBC samples were stratified into high- and low-tumor dependency gene signature score groups based on the median signature score, with subsequent matching to samples possessing available SNV data. Somatic mutation analysis was performed using the maftools package, including identification of differentially mutated genes between groups and visualization of co-mutation and mutual exclusivity patterns.

### Quality control and downstream analysis of scRNA-seq

The entire single-cell RNA sequencing (scRNA-seq) analysis workflow was implemented using the Seurat R package (v5.0.0). Quality control procedures included: 1. Filtering low-quality cells; 2. Doublet removal; 3. Data integration; 4. SCTransform normalization; 5. Batch effect correction; 6. Cell type annotation. The details could be found in our previously published studies. Epithelial cells were subset for downstream analysis. The tumor dependency gene signature score was calculated for epithelial cells using the AddModuleScore() function. Pseudotime trajectory reconstruction was performed on epithelial cells using the slingshot algorithm. Marker genes for epithelial subpopulations were identified with FindAllMarkers() (min.pct = 0.25, logfc.threshold = 0.25). Gene Ontology (GO) enrichment and Hallmark pathway analysis for the C6 subpopulation-specific markers were conducted using clusterProfiler with Benjamini-Hochberg correction.

### CMAP analysis

Potential therapeutic compounds targeting patients with high C6 subpopulation infiltration were identified using the Connectivity Map (CMAP) database. Specifically, the top 150 upregulated genes and top 150 downregulated genes from the high C6 infiltration group were submitted to the platform. CMAP output provides connectivity scores ranging from -100 (maximal antagonism) to +100 (maximal similarity). Compounds with scores ≤ -90 were prioritized as candidates, with lower scores indicating stronger inhibitory effects. The 50 lowest-ranked compounds were selected as potential therapeutic agents for further validation.

### Spatial transcriptomics analysis

The whole downstream analysis was applied by Seurat package. Distinct regions were determined by Principal Component analysis (PCA) and assistance of an experienced pathologist. The delineated tumor regions and gene expression levels were visualized by ggplot2 package.

### Cell culture, transfection and function assays

MDA-MB-231 was obtained from ATCC (American Type Culture Collection, Manassas, USA). Details of cell culture and transfection can be referred to in our previously published research. siRNAs targeting *TONSL* constructs were synthesized by GentleGen (Suzhou, China), and the oligonucleotide sequences are listed in **Supplementary File**: **Supplementary Table S1**. The procedures and details of the colony formation experiment and the CCK-8 experiment are as described in previously published studies (17). Data were presented as mean ± standard deviation (SD), and *p* < 0.05 was considered significant.

## Results

### Identification of tumor dependency genes via genome-wide CRISPR-CAS9 screening

**Figure 1:**
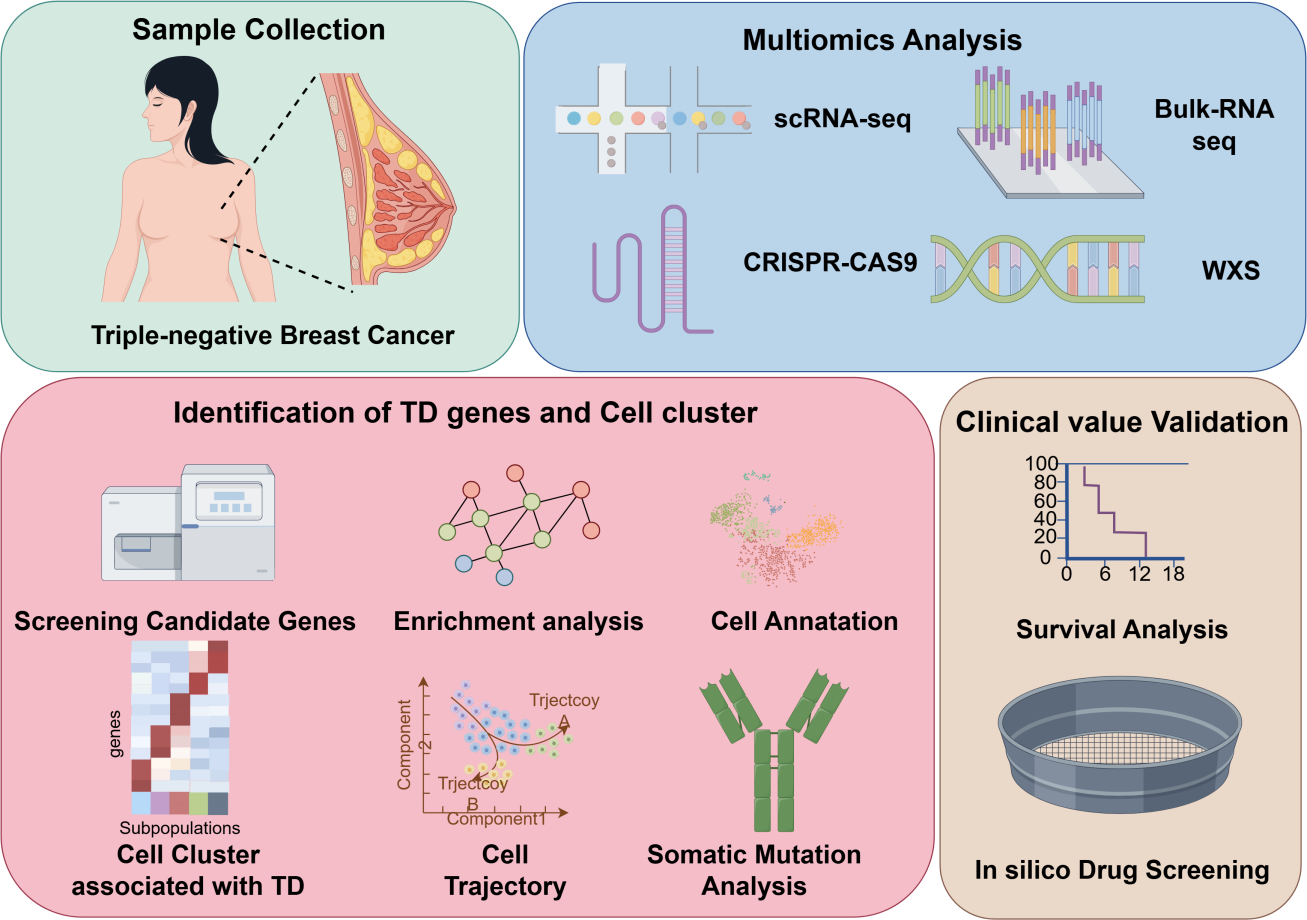
Workflow of the study. TD, tumor dependency; WXS, whole exome sequencing.

**Figure 2:**
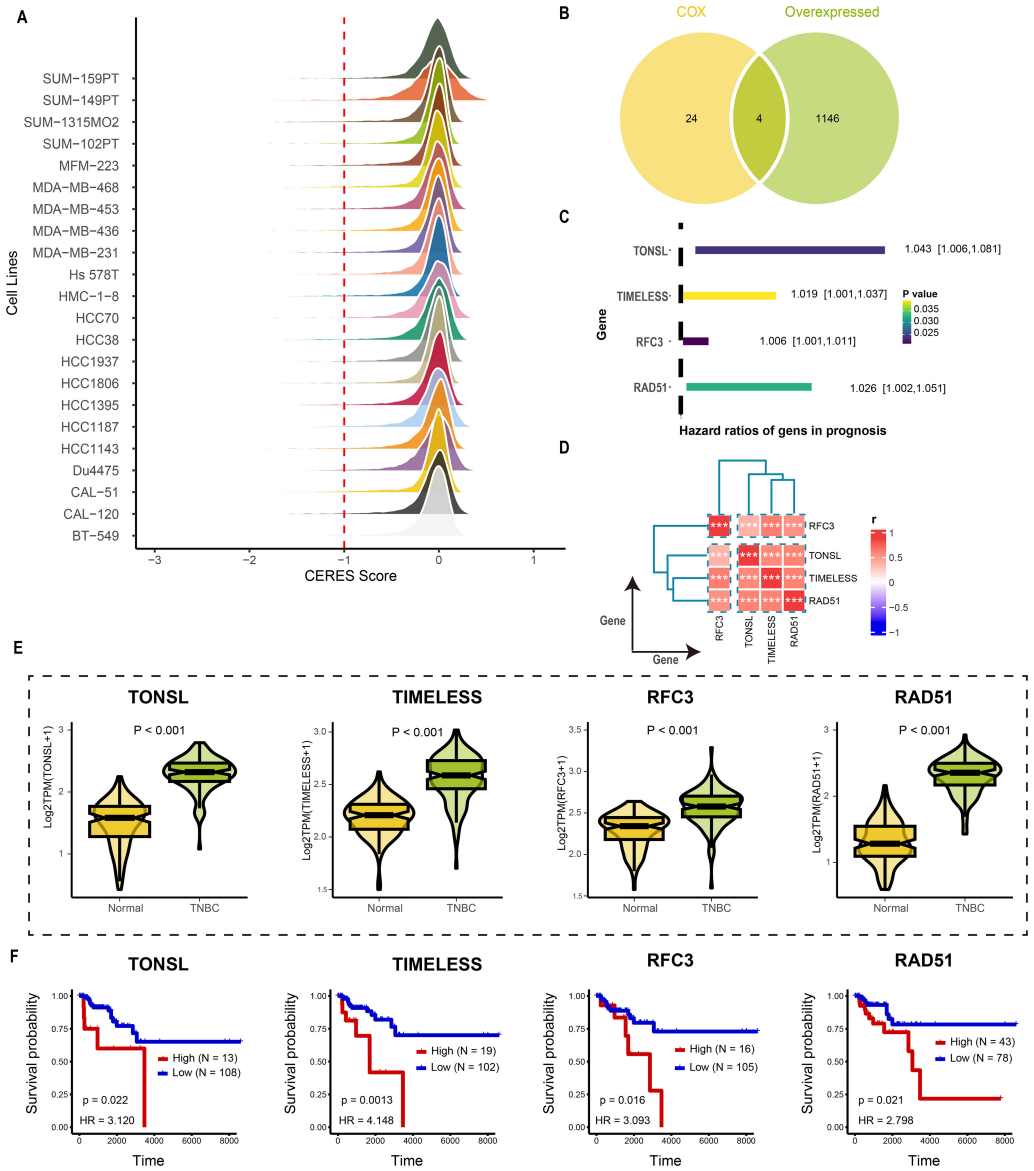
Identification the tumor dependency genes in TNBC. **(A)** The CERES score of candidate genes in TNBC cell lines. **(B)** The venn plot of screening tumor dependency genes. **(C)** The forest plot of tumor dependency genes in TNBC with prognostic value. **(D)** The correlation analysis among tumor dependency genes. ***p < 0.001 **(E)** The expression level of the tumor dependency genes between normal and TNBC tissue. **(F)** The KM plot of the tumor dependency genes.

The overall design of study was illustrated in **Figure 1**. Tumor dependency genes are essential for cancer cell survival and proliferation. Using genome-wide CRISPR-Cas9 knockout data processed by the CERES algorithm from 22 TNBC-like cell lines, we identified 665 tumor dependency genes under stringent criteria (mean CERES score < −1 in ≥80% cell lines; **Figure 2A**). Univariate Cox regression analysis prioritized 28 genes significantly associated with poor prognosis (P < 0.05, HR > 1). Differential expression analysis of TNBC versus normal tissues revealed 1,150 upregulated genes (log_2_FC > 0.5, adj.P < 0.05). Intersection of these gene sets yielded four high-confidence tumor dependency genes (*TONSL, TIMELESS, RFC3, RAD51*) for further investigation (**Figure 2B**: **Supplementary Table S2**). Forest plots confirmed their prognostic risk (**Figure 2C**), while strong inter-gene correlations (P < 0.001; **Figure 2D**) and marked overexpression in TNBC versus normal tissues (**Figure 2E**) underscored their pathological relevance. Critically, Kaplan-Meier analysis demonstrated that high expression of each gene predicted reduced overall survival (log-rank P < 0.01; **Figure 2F**). Collectively, integration of CRISPR-Cas9 functional genomics with transcriptomics defined four TNBC-specific tumor dependency genes that drive poor outcomes, nominating them as potential biomarkers and therapeutic targets.

### Tumor dependency genes were potentially involved in cell proliferation

**Figure 3:**
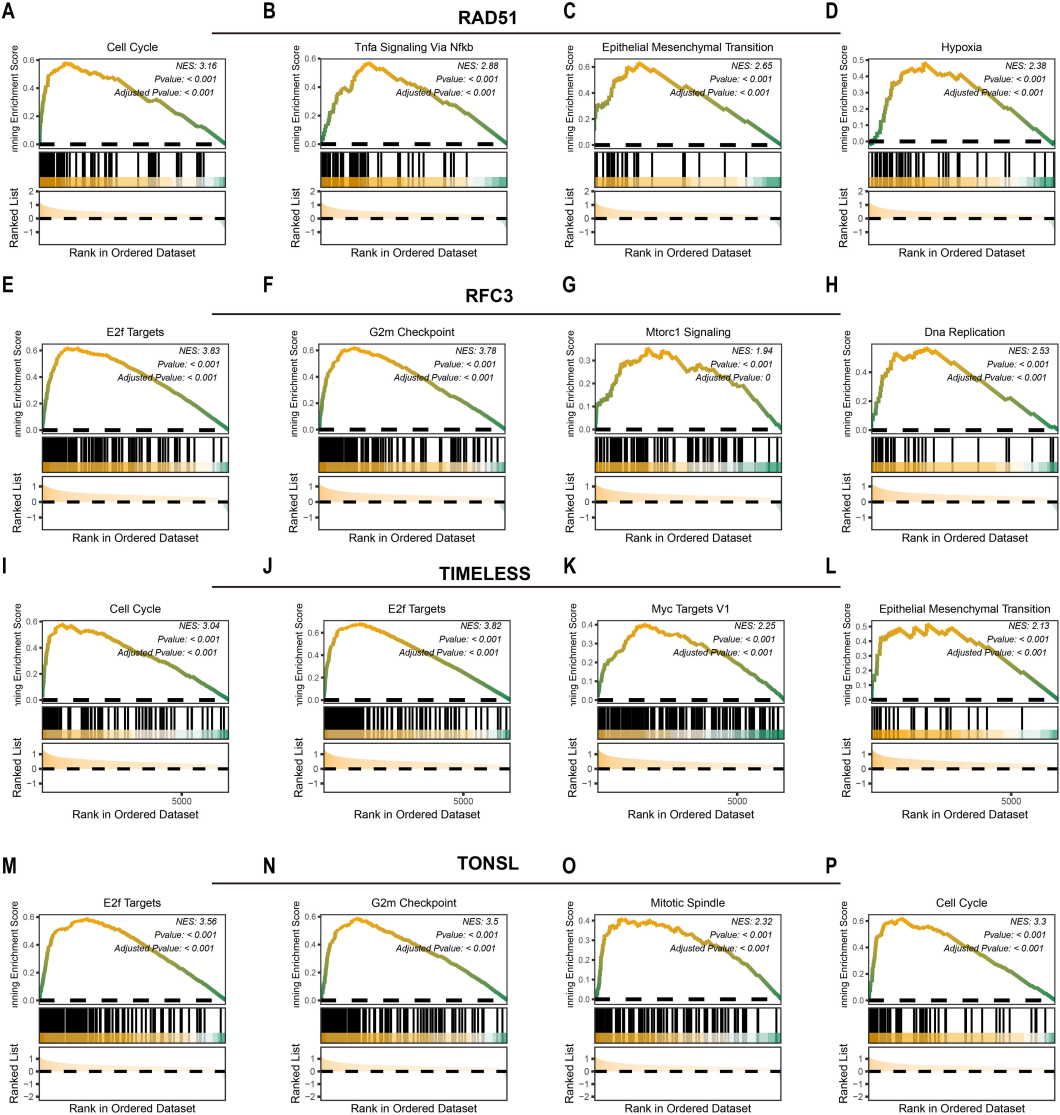
Potential downstream pathways of tumor dependency genes. The potential downstream pathways affected by RAD51 **(A-D)**, RFC3 **(E-H)**, TIMELESS **(I-L)** and TONSL **(M-P)**.

We next investigated downstream pathways potentially regulated by the identified tumor dependency genes. High expression of *RAD51* activated pro-tumorigenic pathways including cell cycle (NES = 3.16, P<0.001; **Figure 3A**), TNFα signaling via NF-κB (NES = 2.88, P<0.001; **Figure 3B**), epithelial-mesenchymal transition (NES = 2.65, P<0.001; **Figure 3C**), and hypoxia response (NES = 2.38, P<0.001; **Figure 3D**). Similarly, elevated *RFC3* expression enriched E2F targets (NES = 3.83, P<0.001; **Figure 3E**), G2/M checkpoint (NES = 3.78, P<0.001; **Figure 3F**), MTORC1 signaling (NES = 1.94, P<0.001; **Figure 3G**), and DNA replication (NES = 2.38, P<0.001; **Figure 3H**). For *TIMELESS*, pathway activation encompassed cell cycle progression (NES = 3.04, P<0.001; **Figure 3I**), E2F targets (NES = 3.82, P<0.001; **Figure 3J**), MYC targets v1 (NES = 2.25, P<0.001; **Figure 3K**), and epithelial-mesenchymal transition (NES = 2.13, P<0.001; **Figure 3L**). Finally, *TONSL* overexpression drove E2F targets (NES = 3.56, P<0.001; **Figure 3M**), G2/M checkpoint (NES = 3.50, P<0.001; **Figure 3N**), mitotic spindle assembly (NES = 2.32, P<0.001; **Figure 3O**), and cell cycle regulation (NES = 3.30, P<0.001; **Figure 3P**). In summary, *RAD51, TIMELESS, RFC3*, and *TONSL* converge on pro-proliferative pathways that sustain tumor survival and expansion.

### C6 subgroup was highly associated with tumor dependency in TNBC

**Figure 4:**
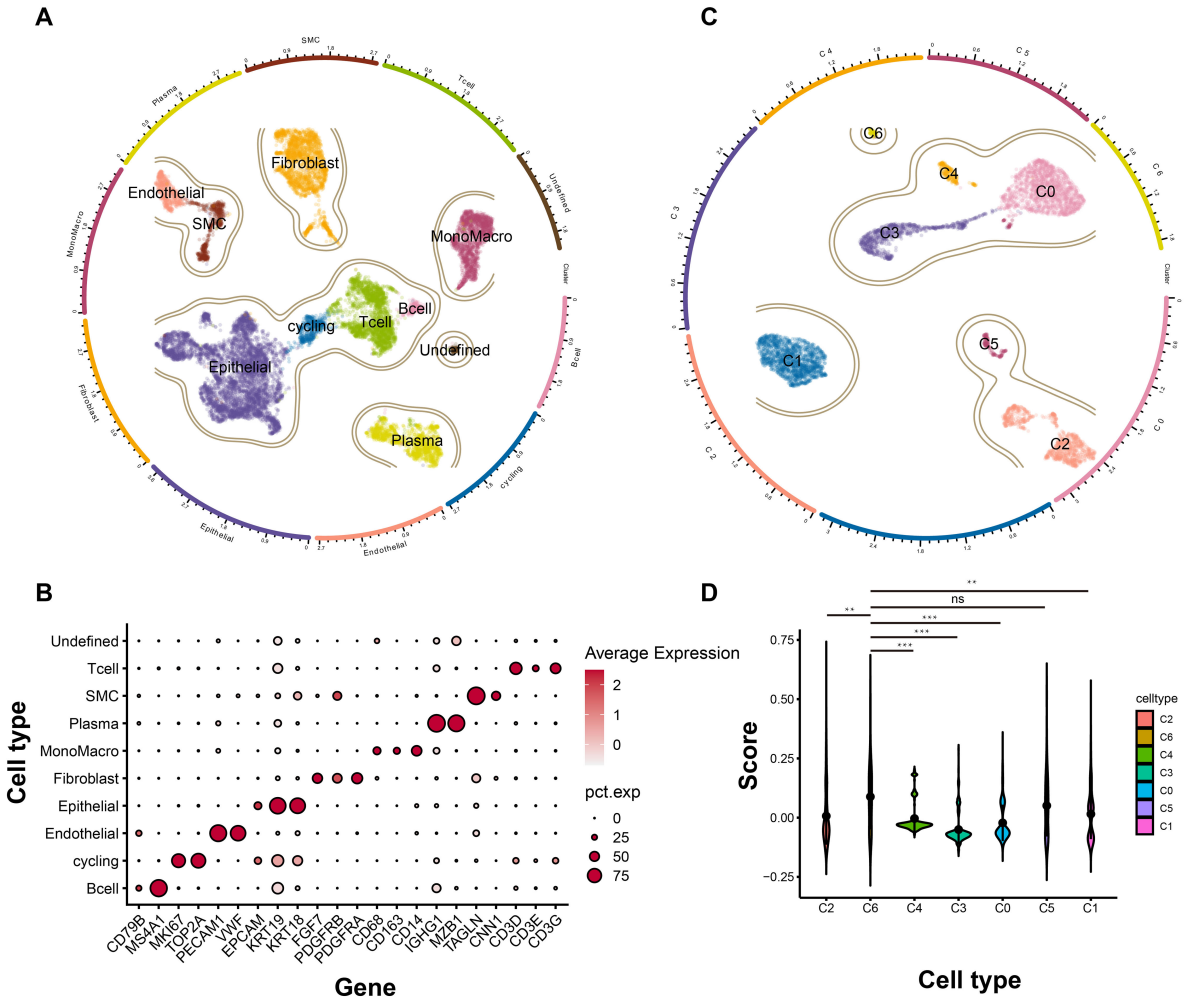
The landscape of TNBC at single cell level. **(A)** The general profile of different cell types in TNBC. **(B)** The expression level of different marker genes corresponding to each cell types. **(C)** The subgroup cell types of epithelial cells in TNBC. **(D)** The signature score of each subgroup based on the tumor dependency genes. ns, no significant; **p < 0.01; ***p < 0.001.

Having identified the core tumor dependency genes, we next characterized their enrichment within epithelial subpopulations in the tumor microenvironment. Integrated analysis of scRNA-seq data from five TNBC samples yielded 15,080 high-quality cells after rigorous quality control and standardized processing (harmony batch correction, SCTransform normalization). Unsupervised clustering resolved nine major cell types and one undetermined population: 254 B cells, 529 cycling cells, 569 endothelial cells, 5,412 epithelial cells, 2,180 fibroblasts, 1,624 monocytes/macrophages, 1,406 plasma cells, 631 vascular smooth muscle cells, 2,331 T cells, and 144 undetermined cells, with UMAP visualization confirming distinct transcriptional profiles across clusters (**Figure 4A**). Specific markers used for cell type annotations were illustrated in **Figure 4B**. Given the epithelial origin of tumor dependency genes, we subset epithelial cells for re-analysis (re-clustering, subpopulation annotation), identifying seven epithelial subsets (**Figure 4C**). Signature scoring of the four tumor dependency genes revealed maximal enrichment in the C6 subpopulation (**Figure 4D**), indicating that C6 epithelial cells exhibit the strongest tumor dependency and likely represent the TNBC compartment most critical for proliferation and survival.

### C6 subgroup was the terminal state of tumor cell differentiation

**Figure 5:**
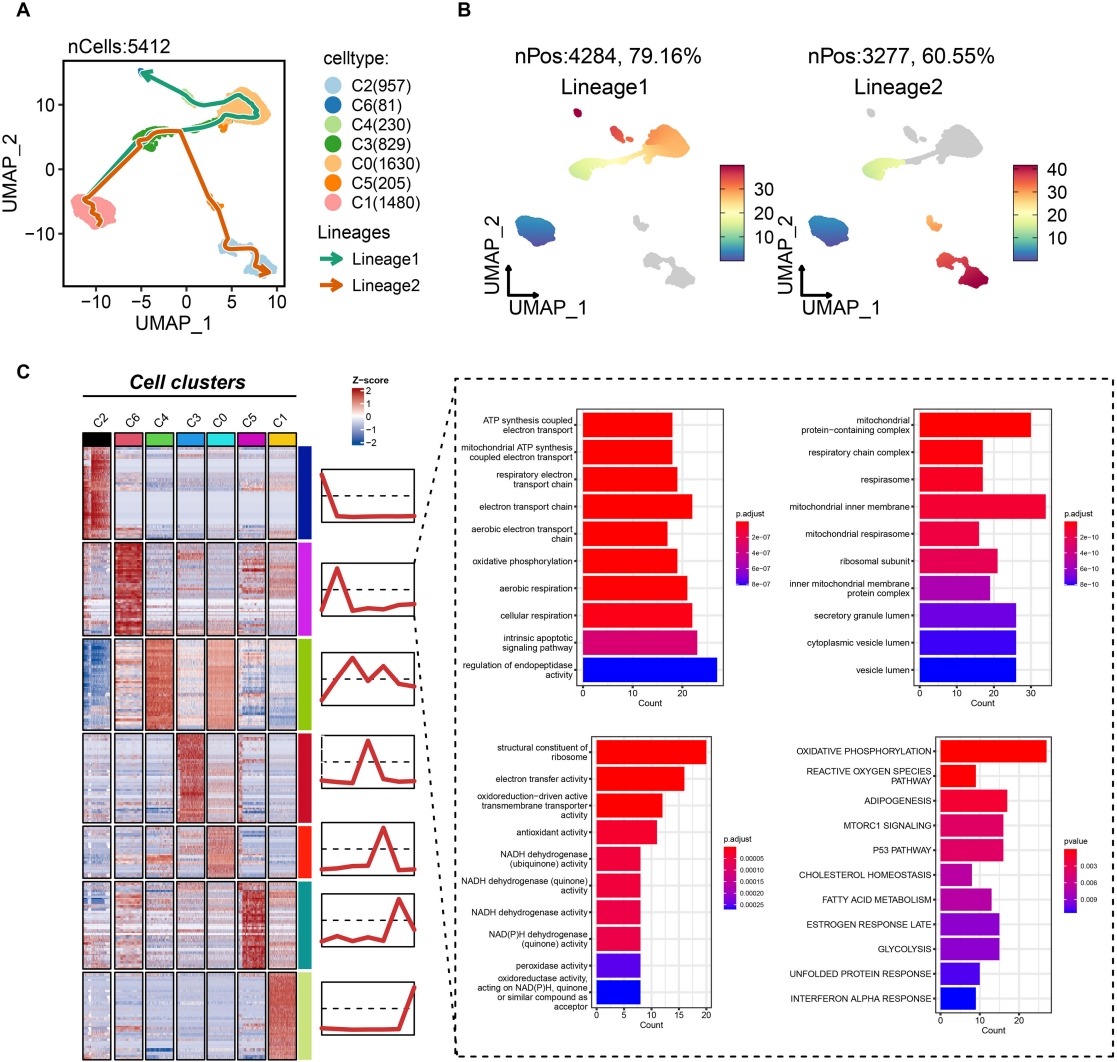
Biological features of the tumor dependency subgroup of epithelial cells in TNBC. **(A)** The pseudotemporal trajectories of epithelial subgroups. **(B)** The cell proportion of two trajectories. **(C)** The biological feature and possibly activated pathway of C6 subgroup.

To delineate the potential role of the C6 subpopulation in disease progression, we reconstructed pseudotemporal trajectories of epithelial cells using the slingshot algorithm. Uniform Manifold Approximation and Projection (UMAP) visualization revealed two principal differentiation paths across 5,412 epithelial cells (**Figure 5A**). Lineage 1 (traversed by 79.6% of cells) originated from C1/C3 subpopulations, progressed through C0/C4 intermediates, and culminated in the C6 terminal state (**Figure 5B**). Conversely, lineage 2 (60.55% of cells) evolved from C1/C3 through C5 toward C2. These trajectories illuminate TNBC epithelial dynamics, positioning C6 as an evolutionarily terminal subpopulation. Differential expression and functional enrichment analyses demonstrated the association of C6 subgroup with mitochondrial bioenergetics, including mitochondrial ATP synthesis-coupled electron transport, electron transport chain, oxidative phosphorylation, respiratory chain complex assembly, and NADH dehydrogenase activity (**Figure 5C**). Notably, proliferative pathways—mTORC1 signaling and p53 pathway—were co-enriched in C6. Collectively, C6 exhibits reprogrammed energy metabolism coinciding with cell-cycle dysregulation, suggesting its pivotal role in driving tumor growth and progression.

### Focal somatic mutations were associated with infiltration level of C6 subgroup

**Figure 6:**
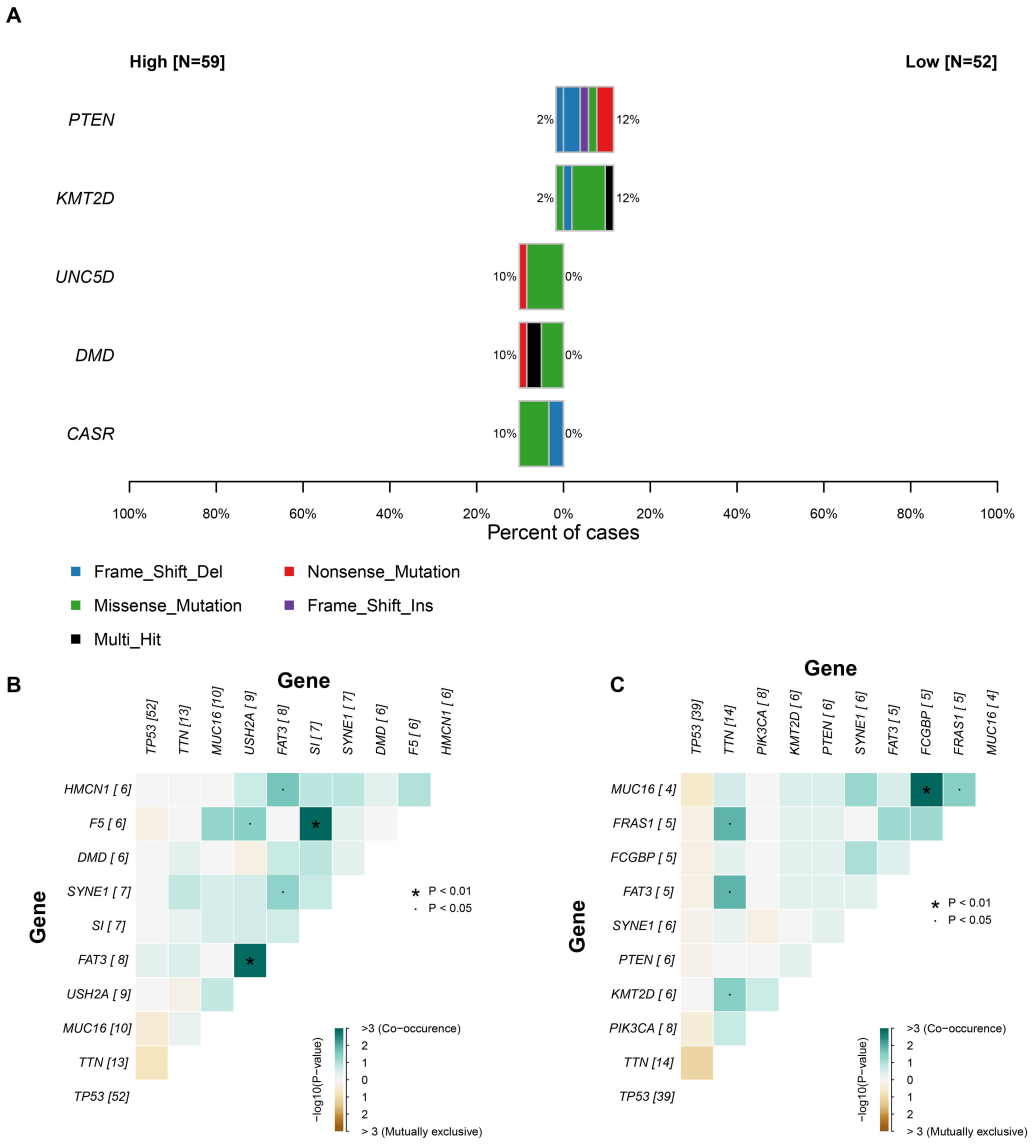
The relationship between somatic mutation and C6 subgroup. **(A)** The significant gene mutation between high and low C6 subgroup. **(B-C)** The landscape of co-mutation in patients with high and low C6 subgroup.

Given the adverse prognostic role of the C6 subpopulation, we investigated factors influencing its tumor infiltration heterogeneity by integrating TCGA transcriptomic and genomic data. Samples with matched datasets were analyzed using the tumor dependency gene signature score as a proxy for C6 enrichment. Fisher’s exact tests identified significantly differential somatic mutations between high- and low-C6 groups: *UNC5D, DMD*, and *CASR* exhibited higher mutation rates in the high-C6 cohort, whereas *PTEN* and *KMT2D* mutations were enriched in the low-C6 group (P<0.05; **Figure 6A**), suggesting somatic alterations may modulate subpopulation abundance. Mutational landscape analysis further revealed distinct co-occurrence patterns between the groups (**Figures 6B, C**), including heterogeneity in co-mutated gene combinations. This demonstrates a nuanced interplay between somatic mutations, tumor dependency, and C6 infiltration dynamics.

### Precision treatment implications based on C6 subgroup abundance

**Figure 7:**
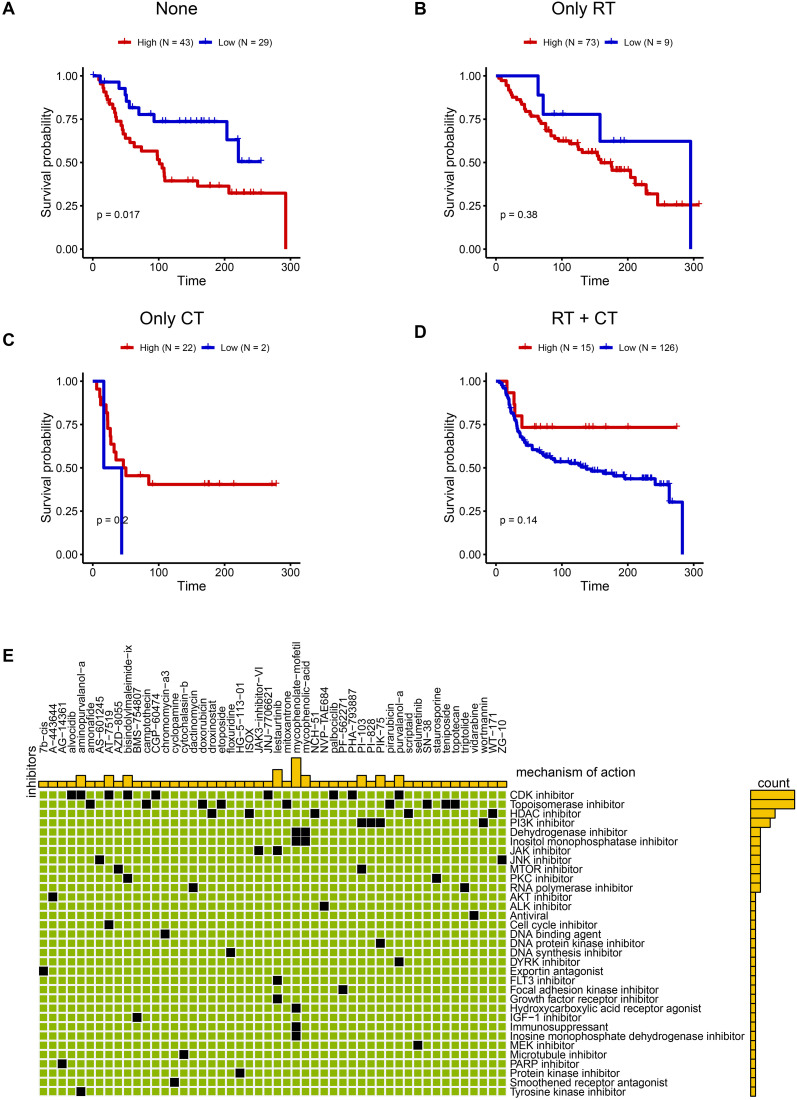
Promising treatment strategies for TNBC patients with C6 subgroup. **(A-D)** The KM plot between high and low C6 subgroup in none of radiotherapy or chemotherapy **(A)** N_high_=43, N_low_ =29), only radiotherapy **(B)** N_high_=73, N_low_ =9), chemotherapy **(C)** N_high_=22, N_low_ =2) and both chemotherapy and radiotherapy patients **(D)** N_high_=15, N_low_ =126). **(E)** The potential drugs for high C6 subgroup.

Building on the malignant functional profile of the C6 subpopulation, we explored personalized therapeutic strategies using transcriptomic and clinical data from 319 TNBC patients in the METABRIC cohort. Single-sample GSEA (ssGSEA) quantified C6 enrichment via the tumor dependency gene signature. Patients were stratified by treatment history: no radiotherapy/chemotherapy (n=72), radiotherapy alone (RT-only, n=82), chemotherapy alone (CT-only, n=24), and combined RT+CT (n=141). Optimal cut-point analysis revealed that high C6 infiltration predicted significantly worse overall survival in patients received only surgery (log-rank *P* = 0.017; N_high_=43, N_low_ =29, **Figure 7A**), whereas no significant association was observed in RT-only (*P* = 0.38, N_high_=73, N_low_ =9, **Figure 7B**), CT-only (*P* = 0.2, N_high_=22, N_low_ =2, **Figure 7C**), or RT+CT (*P* = 0.14, N_high_=15, N_low_ =126, **Figure 7D**) groups. This indicates that surgery-alone is suboptimal for high-C6 patients, while radiotherapy and/or chemotherapy may overcome C6-driven adverse outcomes. To expand therapeutic options, we leveraged the Connectivity Map (CMAP) platform to identify potential inhibitors targeting high-C6 TNBC. Top-scoring compounds (connectivity score ≤ -90) were enriched for CDK inhibitors (**Figure 7E**), mechanistically aligning with pro-proliferative phenotype of C6 subgroup. Collectively, these findings provide a framework for biomarker-guided TNBC therapy.

### Validation of candidate genes in TNBC cell lines

**Figure 8:**
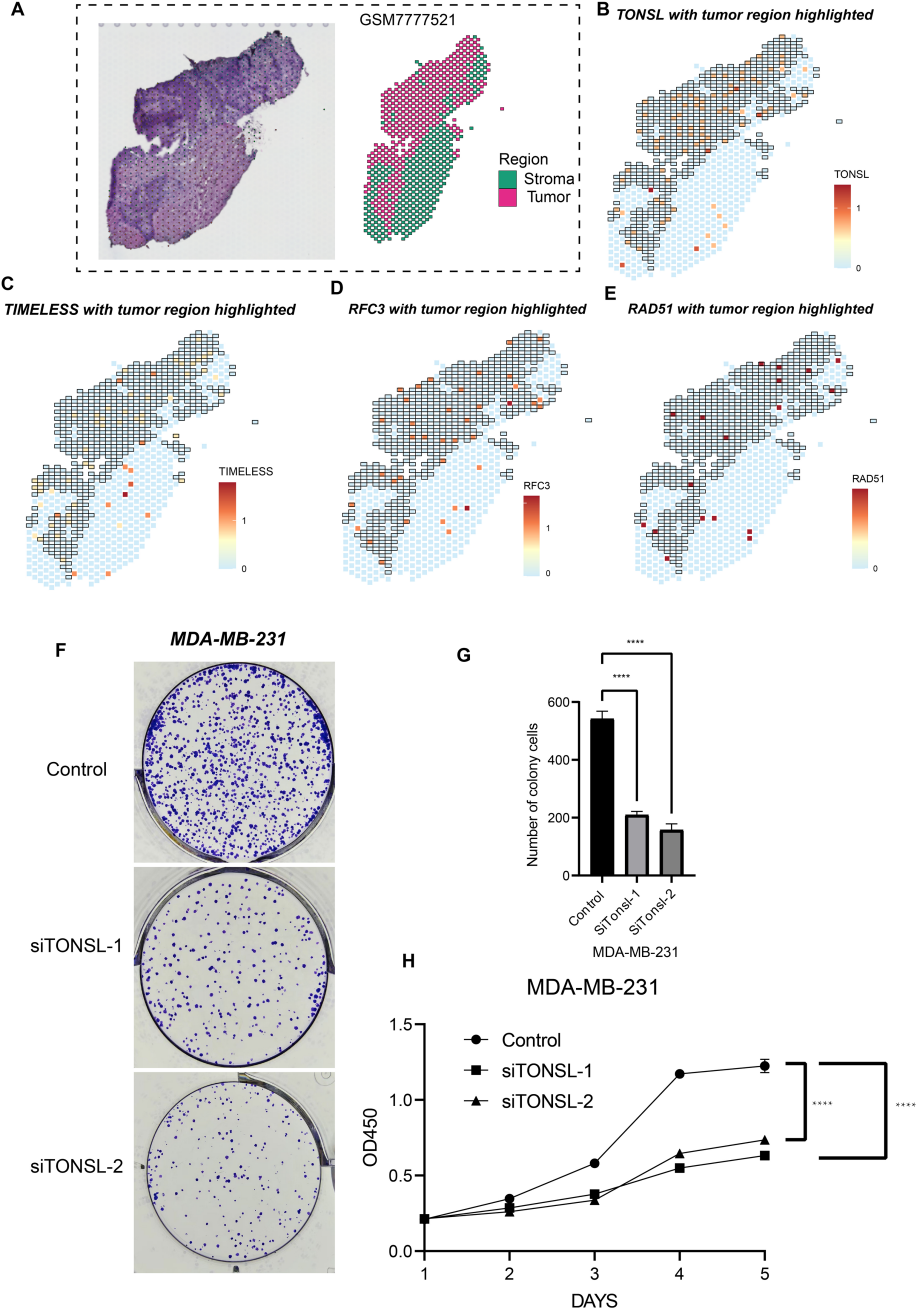
Cell proliferation role of TONSL. **(A)** Sectioning and region delineation of a TNBC spatial transcriptome sample. **(B-E)** The expression of TONSL **(B)**, TIMELESS **(C)**, RFC3 **(D)**, RAD51 **(E)** in spatial transcriptome. **(F-H)** The cell proliferation assays were conducted including colony formation assay **(F-G)** and CCK-8 **(H)**. ****p < 0.0001.

To validate the biological functions and spatial expression patterns of our candidate genes, we analyzed one TNBC sample as illustrated in **Figure 8A**. Dimensionality reduction performed on the spatial transcriptomic profiles identified six distinct regions. Based on differentially expressed genes across these regions, two tumor-enriched areas were delineated. Notably, *TONSL, TIMELESS, RFC3*, and *RAD51* exhibited significantly higher expression levels within the tumor regions compared to the tumor stromal areas (**Figure 8B-E**), suggesting their predominant role in tumor cells. Furthermore, based on prior univariate COX regression analysis, *TONSL* demonstrated the highest hazard ratio. Consequently, we selected *TONSL* for further functional validation. Knockdown of *TONSL* impaired clonogenic formation and proliferative capacity in MDA-MB-231 cells (**Figure 8F-G**). Collectively, these findings indicate that candidate genes including TONSL may represent potential therapeutic targets for triple-negative breast cancer.

## Discussion

During tumor cell proliferation, some genes could play critical role in supporting cell growth. When the expression of these genes is reduced or when they are knocked out, tumor growth is suppressed and may even lead to cell death. In this study, we used the CRISPR-Cas9 system to identify such tumor dependent genes in TNBC cells, including *TONSL, TIMELESS, RFC3*, and *RAD51*. These genes are primarily involved in DNA replication and repair, suggesting that TNBC tumor cells exhibit active DNA replication while potentially experiencing DNA mismatches and damage (18–20). Interestingly, all four genes are also associated with patient prognosis in TNBC. Based on this, we defined a tumor dependency signature by integrating their expression profiles within the TME. Tumor cells exhibiting this high dependency signature may represent a terminally differentiated state and tend to carry more complex somatic mutations, indicating higher genomic instability. Patients with tumors enriched in these high-tumor-dependency cells have worse clinical outcomes. Thus, identifying this cell population through the expression of *TONSL, TIMELESS, RFC3*, and *RAD51*, and targeting them with candidate drugs identified in this study, may offer promising strategies for personalized therapy tailored to different TNBC progression states.

*TONSL* is a key regulator of homologous recombination during chromosomal repair. H4K20me0 is a histone modification associated with post-replicative chromatin. *TONSL* forms a complex with *MMS22L*, which recognizes H4K20me0 and directs the DNA repair machinery to the sites requiring homologous recombination (21). In cancers, this HR function of TONSL has been identified as essential for maintaining cancer stem cell (CSC) properties (22). CSCs are a subpopulation of tumor cells with long-term survival capacity, self-renewal potential, and tumor-initiating ability (23). These findings suggest that tumor cells with high *TONSL* expression may possess stem-like features and that TONSL may play a pivotal role in maintaining CSC characteristics, potentially contributing to poor clinical outcomes in TNBC patients (19). *TIMELESS* is another gene implicated in the DNA damage response. It contributes to genome stability by preventing replication fork stalling at difficult-to-replicate genomic regions (24), potentially through promoting homologous recombination at DNA damage sites with the assistance of PARP-1 (25). Intriguingly, *TIMELESS* also belongs to the family of circadian clock genes, and circadian disruption has been increasingly recognized as a contributing factor in tumorigenesis (26). Similarly, *RFC3* and *RAD51* are involved in the repair of DNA damage (27, 28). In cancers, enhanced DNA repair capacity may indicate resistance to chemotherapy. For example, platinum-based chemotherapeutic agents like cisplatin exert their effects by inducing DNA strand breaks, ultimately leading to tumor cell death due to impaired proliferation (29). However, when tumor cells exhibit robust DNA repair responses, they may evade chemotherapy-induced cytotoxicity, thereby maintain proliferation and promote tumor progression. Moreover, downstream pathways associated with these four genes—including mTORC1, E2F, and EMT—have been identified as critical drivers of tumor progression and clinical deterioration (30–32). Therefore, our results suggest that these four candidate genes, *TONSL, TIMELESS, RFC3*, and *RAD51*, may contribute to TNBC progression by participating in DNA damage repair and by activating oncogenic signaling pathways.

In TNBC tissues, we identified an epithelial cell subpopulation with high expression of four tumor dependent genes using single-cell RNA sequencing. As described above, these cells exhibit high proliferative capacity and active DNA repair pathways. Interestingly, this subpopulation is located at the terminal end of the epithelial cell differentiation trajectory, suggesting that these cells may represent terminally differentiated tumor cells within the TNBC. Notably, these cells also display upregulation of mitochondrial function-related pathways, such as oxidative phosphorylation and electron transport chain activity, indicating that mitochondrial energy metabolism is highly active in this subpopulation. Typically, tumor cells rely predominantly on anaerobic glycolysis for energy production—a phenomenon known as the Warburg effect (33). However, this highly tumor dependent subpopulation appears to utilize aerobic respiration as its major energy source, implying the presence of metabolic reprogramming. During tumorigenesis and progression, cancer cells often adjust their metabolic profiles in response to changes in the TME and therapeutic pressure (34). Previous studies have shown that oxidative phosphorylation–dominant metabolism can activate TGF-β and MAPK signaling pathways, thereby enhancing the epithelial-mesenchymal transition (EMT) process and promoting tumor metastasis (35). Moreover, activation of oxidative phosphorylation suggests sustained mitochondrial function, which may suppress mitochondrial membrane permeabilization and other mitochondria-dependent apoptotic pathways (36). This metabolic shift is also critical in cancer stem cells, as it may reinforce stem-like properties and confer resistance to conventional therapies (37). Some studies have further suggested that enhanced oxidative phosphorylation is associated with altered mitochondrial dynamics, such as mitochondrial fragmentation, which could contribute to increased tumor cell motility, metastasis, and disease progression (38). Thus, the TNBC epithelial cell subpopulation with high expression of tumor dependent genes may drive tumor progression through aberrant metabolic reprogramming. In addition, we explored the relationship between these cells and somatic mutations in TNBC. *UNC5D* is a well-known tumor suppressor gene, whose normal expression can inhibit tumor progression (39). Similarly, mutations in *DMD* have been implicated in the development of various cancers (40). In patients with high expression of the four tumor dependent genes, multiple coexisting genetic alterations were frequently observed. These findings suggest that although these genes contribute to enhanced DNA repair capacity in tumor cells, specific mutations may still persist and actively support the malignant phenotype.

To further explore the clinical utility of tumor dependency genes in TNBC patients, we stratified individuals based on a tumor dependency signature score composed of the four key genes. As expected, patients with a high signature score exhibited significantly worse prognoses. Notably, this poor prognosis was independent of whether the patients had undergone surgical treatment. This suggests that, even after thorough surgical resection, patients with high signature scores may still experience events leading to reduced survival quality or other adverse outcomes. Interestingly, for patients who received chemotherapy or radiotherapy following surgery, prognosis was partially improved, and this effect was more pronounced in those who underwent both chemotherapy and radiotherapy. These findings indicate that multimodal therapy may benefit high-score TNBC patients. Based on this stratification, we further conducted drug screening to identify candidate compounds for personalized treatment in patients with high signature scores. Notably, inhibitors targeting pathways such as mTOR, PI3K, and the cell cycle/DNA replication machinery may act downstream of *TONSL, TIMELESS, RFC3*, and *RAD51*. These pathways have been widely recognized for their crucial roles in tumor progression (41–43). Therefore, the use of the tumor dependency score to identify high-risk TNBC patients and guide the administration of targeted therapies may offer a promising approach for personalized cancer treatment.

Despite our exploration of tumor proliferation dependency genes in TNBC, several limitations remain to be addressed. First of all, the criteria on which we screen candidate genes are relatively strict. Although this can find the universality of tumor-dependent genes, it may lead to the filtering of some genes that play an important role in specific TNBC subtypes. This study only preliminarily confirmed the effect of TONSL on the phenotype of tumor cells. The other candidate genes RFC3, TIMELESS, and RAD51 have not been confirmed yet, which is also a limitation of our research. Additionally, the predicted downstream pathways of these genes require further validation through both *in vitro* and *in vivo* experiments to clarify their roles in tumor progression. Given our hypothesis that the tumor dependency signature composed of these four genes may stratify TNBC patients and guide personalized management based on prognosis, future research should assess the stratification performance in large-scale clinical trials. It is worth noting that our exploration in the spatial transcriptome data was based on only one sample, which may lead to bias in the results. More samples need to be included in the future to confirm the universality of our conclusion. Finally, the candidate compounds identified for TNBC patients with high tumor dependency scores need to be tested in preclinical models to confirm their efficacy in inhibiting tumor proliferation and metastasis.

## Conclusion

Overall, our study identifies *TONSL, TIMELESS, RFC3*, and *RAD51* as tumor dependency genes. These genes may contribute to TNBC progression and poor prognosis by promoting tumor growth and protecting cancer cells from chemotherapy-induced damage. Furthermore, the signature score derived from these genes may aid in stratifying TNBC patients based on disease progression and offer a potential framework for personalized therapeutic strategies.

## Data Availability

The original contributions presented in the study are included in the article/**Supplementary Material**. Further inquiries can be directed to the corresponding author.
